# Increasing access to self-managed abortion through pharmacies: programmatic results and lessons from a pilot program in Oromia, Ethiopia

**DOI:** 10.3389/frph.2025.1472696

**Published:** 2025-03-04

**Authors:** Bekalu Mossie Chekol, Abiyot Belai Mehari, Blain Rezene, Samuel Muluye, Yadeta Ayana, Elsabet Sisay, Sally Dijkerman, Genene Assefa

**Affiliations:** ^1^ Ipas Ethiopia, Addis Ababa, Ethiopia; ^2^ Oromia Health Bureau, Addis Ababa, Ethiopia; ^3^ Ipas United States, Durham, NC, United States

**Keywords:** abortion, comprehensive abortion care, Ethiopia, self-managed abortion, self-care, private pharmacy, medical abortion

## Abstract

Despite increased availability of safe abortion following legal reform in Ethiopia, one-half of public sector abortion services are treatment of postabortion complications, indicating challenges meeting women's needs. Self-managed abortion (SMA)—the ability of pregnant people to manage their unwanted pregnancies with or without the support of a health care provider—is a safe, feasible, and acceptable option for women at gestational ages up to 12 weeks. Seeing the potential of SMA to vastly expand access to safe abortion and reduce postabortion complications, a pilot initiative targeting private pharmacies was implemented by the Ethiopian Ministry of Health, the Oromia Regional Health Bureau, and a non-profit organization. From December 2021 to March 2023, implementers trained and supported 41 pharmacies to provide SMA counseling and medical abortion drugs, with and without prescriptions, which was considered to contradict the legal framework at the time. Pharmacy clients' SMA experiences were documented in logbooks and via 21-day follow-up phone surveys. Thirty-two pharmacies (78%) supported 1,457 self-managed abortions during the pilot. Among clients with complete follow-up surveys (*n* = 1,233), 98.3% had a complete abortion without needing additional treatment. Only four clients (0.3%) reported a complication. The pilot demonstrated high demand for and feasibility of increasing access to quality SMA through private pharmacies in Ethiopia, but challenges remain due to the lack of a legal framework. We recommend providing multi-sector support on SMA to private pharmacies so they can in turn improve safe abortion accessibility by bringing safe, acceptable services closer to the people that need them.

## 1 Introduction: problem being addressed and rationale for the proposed innovation

### 1.1 History and legality of medical abortion in Ethiopia

Maternal mortality from unsafe abortions has significantly decreased in Ethiopia since the reform of the abortion law in 2005 (1). This decline has been attributed to the development of national safe abortion technical and procedural guidelines, integration of comprehensive abortion care (CAC) in public health facilities, and task sharing of abortion provision with mid-level providers (2). The expansion of medical abortion (MA) availability through these strategies played a significant role in increasing access to safe abortion nationally. Medical management of induced abortion using misoprostol alone or with a combined regimen of mifepristone and misoprostol, henceforth called MA, is recommended by the World Health Organization (WHO) due to its high efficacy. With Ethiopia's 2005 legal reform and subsequent safe abortion guidelines in 2006, mifepristone was legalized for safe abortion, and MA was formally introduced in the country in 2019. Notably, Ethiopia was among the first wave of countries to grant regulatory approval for a mifepristone and misoprostol combined regimen called Medabon. Initially, however, MA was limited to use up to nine weeks of gestation (3). A 2014 revision of the national guidelines expanded MA use beyond nine weeks gestation in line with WHO recommendations (2, 4).

Following the introduction of MA in Ethiopia, the share of induced abortions performed in public and private health facilities using medication increased to over one-third (35.6%) by 2014 (5). Since then, the WHO updated its guidelines in 2022 to expand their recommendations to include MA provision by pharmacists, pharmacy workers, and pregnant people themselves (6). However, at the time of this paper's case study, MA provision in the country was still restricted to health providers only, excluding pharmacists and pregnant people (2, 4). The regulatory guidelines allowed pharmacists to fill prescriptions for MA drugs, but they were prohibited from providing MA drugs without a prescription.

### 1.2 Increasing rate of abortion complications in Ethiopia

**Figure 1:**
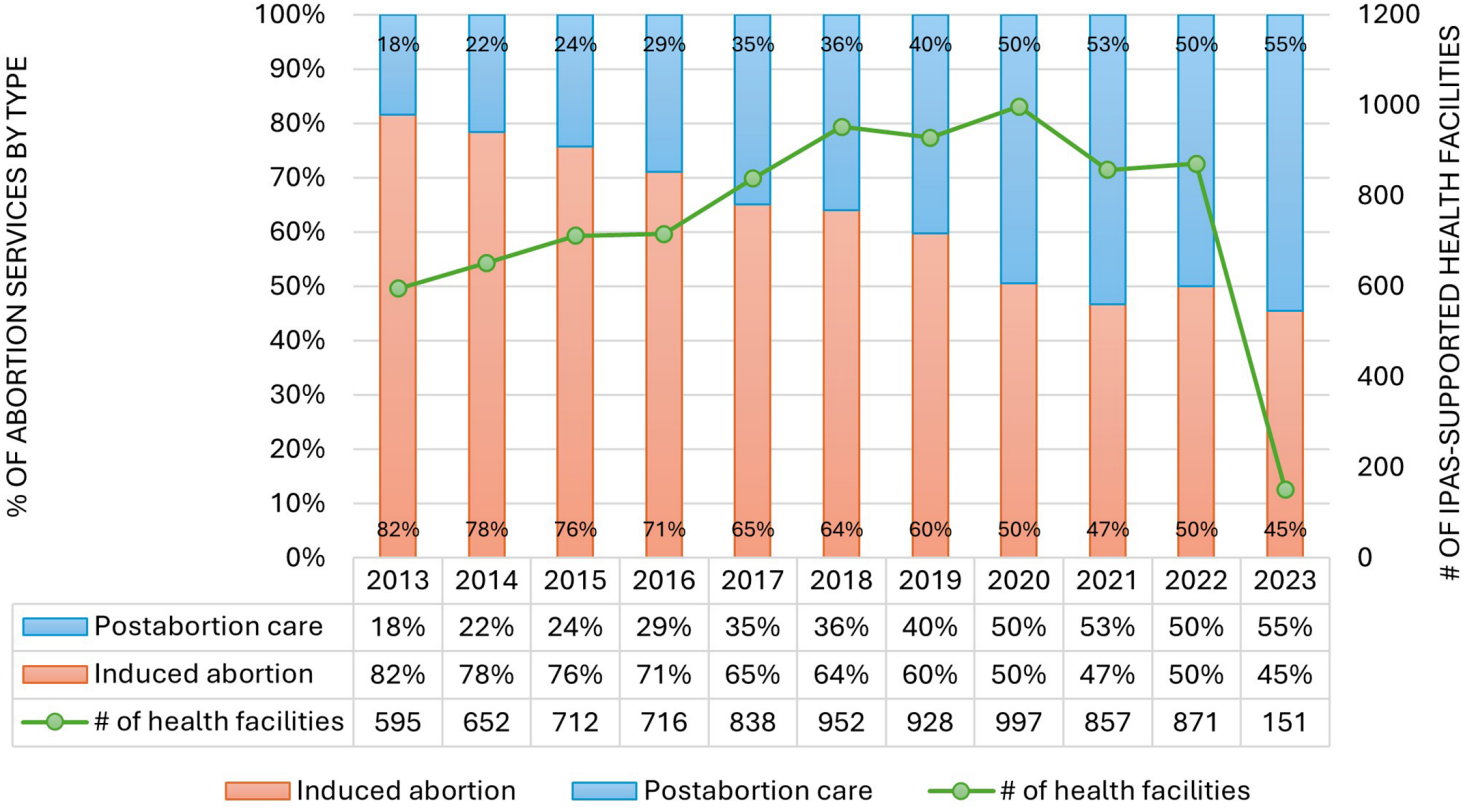
Annual abortion services by type in ipas-supported health facilities.

Despite the vast expansion of induced abortion service availability in health facilities, the volume of postabortion complications, called postabortion care (PAC), remains close to 50% of CAC services being provided in the public sector, suggesting continued difficulties in eliminating unsafe abortions in the country (7). In fact, recent public sector logbook records indicate that the proportion of abortion services that are PAC is on the verge of outpacing induced abortion services. Monitoring data from health facilities supported by Ipas, an NGO that supports public health facilities to provide and monitor high quality comprehensive abortion care, clearly demonstrates this shift (Figure 1). Similarly, a national study found that the number of people receiving treatment in a health facility for complications from an abortion (performed in or outside a health facility) nearly doubled between 2008 and 2014, rising from 52,600 to 103,600 cases. Fifteen percent of people seeking abortion care at health facilities reported trying to end the pregnancy themselves (8).

With a goal to understand the underlying causes of the rise in PAC, a subset of the authors of this paper conducted exploratory research on patients' pathways to public sector abortion care and their perspectives on the quality of care they received. Better accessibility of private providers, timeliness of care, and ensuring privacy and confidentiality were among the key reasons that people decided to seek abortion services from private providers and pharmacies. Unfortunately, most patients reported that they did not receive enough information on how to administer the medical abortion drugs, the side effects, how to identify complications, and when and where to seek additional care (9). Accessing medical abortion through private pharmacies is theorized by local experts to account for a substantial number of PAC cases presenting in the public health system, yet these complications are largely preventable. Although not legal, MA is widely accessible outside of health facilities, so people continue to get the MA drugs from pharmacies and other informal sources to end their pregnancies.

### 1.3 Rationale for proposed intervention: increasing access to safe abortion through private pharmacies

Self-managed abortion (SMA) is the ability of pregnant individuals to manage their unwanted pregnancies with or without the support of health care providers, particularly in the early weeks of pregnancy (up to 12 weeks' gestation). WHO defines SMA as “self-management of the entire process of medical abortion or one or more of its component steps, such as self-assessment of eligibility for medical abortion, self-administration of medicines without the direct supervision of a health worker, and self-assessment of the success of the abortion process” (pg. xvi in *Abortion Care Guidelines*) (6). Research has proven self-managed abortion to be safe, feasible, and acceptable by pregnant persons with gestational ages up to 12 weeks, indicating its potential to vastly expand access to timely, high quality, safe abortion care (6).

Evidence suggests that SMA is on the rise globally, owing to the availability of MA, a non-invasive, safe, and simple method of terminating a pregnancy. For instance, in a study conducted in India, of the estimated 15.6 million abortions conducted in 2015, 73% were performed using MA outside of a health facility (10). SMA has many positive attributes, including high acceptability among users due in part to its promise of heightened autonomy, confidentiality, as well as being less invasive than surgical methods (11). A randomized controlled trial in Indonesia comparing medical abortion counseling provided through a safe abortion hotline vs. standard of care found no differences in clients' feelings of support and preparedness for SMA (12). A mixed methods evaluation of an SMA program using community agents in Bolivia found that 99% of people successfully self-managed their abortion (13). Research on SMA through telemedicine in the US found similarly high success rates (96%) and patient satisfaction (98%) (14).

A non-randomized, non-inferiority study in Ghana compared self-reported clinical outcomes following medical abortion sourced from either a pharmacy or clinic, finding similar levels of client adherence to the abortion regimen and low levels of additional care needed (2.9% in the pharmacy group vs. 4.8% in the clinic group). The authors found that MA drugs accessed directly from pharmacies, without prior consultation of another provider, resulted in non-inferior clinical outcomes compared to clinic-based services (15). A prospective cohort, non-inferiority study in Cambodia comparing medical abortion through pharmacies and clinics similarly found high rates of regimen adherence (above 96% taking the MA pills correctly) and comparable clinical outcomes across the two groups (16). In Nepal, a noninferiority study demonstrated that women receiving MA through trained auxiliary nurse-midwives at pharmacies experienced similar clinical outcomes to those receiving services at a government-certified health facility, concluding that pharmacy-based services are effective and safe (17). Supporting communities to access high quality MA drugs, information, and counseling through pharmacies is therefore essential to extend the reach of the public health system, reduce postabortion complications, and meet the needs of pregnant people.

To further assess the need for the pilot initiative, Ipas conducted a rapid assessment of 41 pharmacies in Adama and Sashamene towns using pharmacist interviews. The assessment documented a significant number of women visiting pharmacies to obtain information and counseling on how to end their unwanted pregnancies. Pharmacists estimated an average demand for MA of 10 women per month (min: 5; max: 25). Most reported that women procured MA drugs from their pharmacy and then took it at their home to terminate their pregnancies. In this community case study, we describe the components, results, and lessons learned from a pilot intervention to increase access to self-managed abortion through private pharmacies in two towns in Oromia, Ethiopia.

## 2 Context: setting and population

The pilot initiative was conducted in Adama and Shashemene towns of Oromia regional state from December 2021 to March 2023. Oromia is the largest regional state in Ethiopia in terms of both population and geographic area. The 2019 Ethiopian Demographic and Health Survey (DHS) estimated a total fertility rate of 4.3 and a modern contraceptive prevalence rate (CPR) of 38.9 (18). As of 2014, the pregnancy rate in Oromia was 249, with an estimated 39% of pregnancies being unintended. Researchers estimated an abortion rate of 9.8% and 37% of abortions being performed outside of health facilities (8). Also in 2014, Oromia had 241 health facilities offering first trimester safe abortion services and PAC (7). Despite the large volume of health facilities offering this care, the treatment rate for abortion complications increased from 3.0 in 2008 to 4.7 in 2014, representing a 58% increase (8).

Pharmacists were key stakeholders in the pilot program. To become a pharmacist in Ethiopia, individuals must obtain a four-year bachelor's degree in pharmacy from an accredited university. To obtain a pharmacist license, they must subsequently pass the Ethiopia National Licensing Examination for Pharmacists and register with the Ethiopian Food and Drug Authority (EFDA). This is followed by continuous professional development to maintain competency and periodic license renewal.

## 3 Key programmatic elements and results

### 3.1 Self-managed abortion through private pharmacies intervention model: method and materials

The pilot initiative was funded by the Packard Foundation under a larger award to Ipas called “*Bridging the Gaps in Abortion Care and Contraceptive Services in Ethiopia*” that ran from October 2020 to March 2023. It began with a Memorandum of Understanding signed by Ipas, the Ministry of Health, and the Oromia Regional Health Bureau (RHB). Following the agreement, consecutive meetings were conducted with RHB officials to elaborate the design, rationale, and the roles of Ipas and the RHB, leading to an agreement whereby Ipas and the RHB would jointly oversee the pilot and together generate evidence to inform national programming. All parties agreed to proceed with the pilot initiative in the absence of a clear, supportive legal framework. These discussions cascaded to zonal officials and regulatory bodies to engage them early in the implementation process and solicit their support for the pilot intervention.

Private pharmacies in the selected towns were eligible to join the pilot if they currently dispensed mifepristone and misoprostol (MA drugs) and were willing to continue doing so throughout the pilot period. To ensure that the pharmacies had access to MA commodities through a preexisting relationship with a supplier, zonal authorities recruited pharmacies from a procurement list obtained from DKT, an international organization supporting the MA supply chain in Ethiopia and many other countries. After an initial training of pharmacists in Adama town, program implementers realized that the selection procedure needed to be more thorough to ensure recruitment of pharmacists most likely to participate in the pilot post-training. They therefore developed a pharmacist screening tool to assess eligibility, which included:

1. Willingness of the pharmacy owner/pharmacist to participate in the pilot training.
2. Willingness of the pharmacy owner/pharmacist to provide MA services with and without prescription.
3. Previous experience of the pharmacy owner/pharmacist in dispensing MA drugs.
4. Evidence of demand for MA services at the pharmacy in the form of recent MA sales data.
5. Pharmacy authorization to stock MA drugs provided by the Drug Administration agency.
6. Self-reported positive abortion values and attitudes by the pharmacy owner/pharmacist.

This selection process was made jointly with the zonal health officials and regulatory bodies from the East Shoa health department. Interested pharmacists reported that a primary reason for participating in the pilot was to receive clarification on MA services, as there was a lot of ambiguity surrounding regulatory issues, leaving them without the necessary knowledge to deliver the service despite the demand at their pharmacies. In addition, the Regional Health Bureau's participation in the pilot gave them confidence to continue providing MA services openly.

Over the course of three training sessions (two in Adama and one in Shashemene), a total of 41 pharmacists received training to provide MA drugs with and without a prescription up to 9 weeks gestational (by date of last menstrual period). To demonstrate support for the pilot initiative, representatives from the Zonal Health Office participated in opening and closing remarks during the training. The objectives of the training were to equip participants with accurate knowledge of MA administration, including both misoprostol-only and combination mifepristone and misoprostol regimens,^1^ and postabortion family planning (PAFP) provision and referrals. Pharmacists were also trained on contraindications for MA, including ectopic pregnancy, allergy or hypersensitivity to mifepristone and/or misoprostol, severe anemia, bleeding disorders, intrauterine contraceptive device in-place, undiagnosed/unresolved uterine abnormality, presence of active infection/sepsis, renal or hepatic insufficiency, chronic adrenal failure, high blood pressure, and current or suspected breast cancer. Additionally, pharmacists were trained to counsel clients on how to recognize the signs of a complete abortion, side effects, danger signs of a complication, and when to seek further medical attention. For additional clinical guidance regarding MA for service providers, please see Clinical Updates in Reproductive Health—Ipas).

Pharmacists were trained to use Hesperian's Safe Abortion App^2^ to estimate clients' gestational age by last menstrual period (LMP) and make use of the application's reference materials. The Hesperian Safe Abortion App is a free mobile and browser application with offline capability available in 11 languages, including Amharic and Afan Oromo. The app provides descriptions of safe abortion methods, correct dosing for medical abortion, how to identify and respond to warning signs, and how to prepare for safely managing an abortion using a checklist (20). Pharmacists were also trained to inform clients about the potential health consequences of providing inaccurate information. If there was any doubt about the accuracy of the information provided by a client, or if the client was unsure of the date of their last menstrual period, the pharmacist referred the client for an ultrasound to confirm the gestational age before providing MA drugs.

The trainings also included activities to support pharmacists in articulating their values, beliefs and attitudes on abortion using Ipas's Values Clarification and Attitudes Transformation (VCAT) toolkit, as well as activities to build their counseling skills to provide compassionate and respectful care. The Ipas VCAT Toolkit is a social and behavioral change communication tool which is designed to help individuals explore their values, beliefs, and attitudes about abortion, fostering greater understanding, empathy, and change in how they view abortion service and women who seek care (21). For more information about VCAT, see Abortion values clarification for action and transformation (VCAT)—Ipas.

Each trainee received a registration logbook chart, which was used to record information about SMA clients and services offered post-training. Pharmacists were instructed to make follow-up calls to consenting clients after 21 days and received 100 Ethiopian Birr per client to document clinical outcomes, including side effects, complications, and any referrals, also recorded in the logbook. Moreover, pharmacists were provided information, education, and communication (IEC) materials such as client brochures in two local languages (Amharic and Afan Oromo) (Supplementary Material 1); these IEC materials informed clients about the expected symptoms and side effects of MA and how to identify danger signs of complications.

Monitoring and evaluation (M&E) experts from the RHB and Ipas, including the authors of this paper, were actively involved early in the program to design the M&E plans and tools for the project and to participate in supportive supervision visits to participating pharmacies. Details of the M&E plan are summarized in Table 1 and included follow-up calls by a member of the M&E team to a randomized subset of consenting MA clients to complete a structured questionnaire. The purpose of these additional follow-up surveys was to confirm accuracy of the pharmacy logbooks and to collect additional information on MA clients' experiences and perceived quality of care.

**Table 1:** Components of project monitoring and evaluation.

Data source	Tool	Purpose	Data collection procedure	Sample description
Pharmacy	Registration logbook chart	Monitor MA caseload volume and client characteristics	Completed after each MA service by pharmacist	*N* = 1,457 MA services (502 in Adama, 955 in Shashemene) provided at 32 pharmacies (16 in Adama, 16 in Shashemene)
Pharmacist	Supportive Supervision Checklist	Provide structured support to the pharmacist, including assessing MA counseling and documentation practices, drug supply, and identifying gaps and corrective measures where needed	Completed at quarterly in-person visits; jointly administered by Ipas Program Coordinators and RHB representatives	Findings from supportive supervision visits to all pharmacies were documented in 2 summary reports by program implementers (August 2022 and May 2023)
MA clients	21-day follow-up call	Document clinical outcomes, including side effects, complications, and any referrals	Verbal consent obtained by pharmacists & recorded in locked logbook; follow-up calls made by pharmacists and results recorded in logbook	*N* = 1,360 (93%) MA clients consented to phone call; *N* = 1,233 (90%) completed follow-up call
MA clients	Structured follow-up questionnaire	Understand MA clients’ experiences in more detail and their perceived quality of care. Check the accuracy of pharmacist logbooks.	Randomly selected subset of consenting MA clients were called and recruited to participate in a phone-based structured questionnaire with a member of the Ipas M&E team.	*N* = 64 selected randomly from logbooks, 2 per pharmacy; *N* = 56 completed (88%) the structured questionnaire (30 in Adama, 26 in Shashemene)

One month after the initial training in Adama Town, program coordinators employed by Ipas and health systems coordinators from the Oromia Regional Health Bureau began providing supportive supervision to pharmacists through monthly in-person visits and weekly phone calls to oversee the progress of the SMA initiative, identify challenges, check on documentation practices, and provide technical and programmatic support to pharmacists. They used a semi-structured supportive supervision checklist to document and provide structured support to the pharmacists. During the first round of support visits, they discovered that just 3 of the 16 trained pharmacies were providing the service, with a lack of MA drug supply serving as the primary factor. Some pharmacists were unsure of where to request the drug, and others were hesitant to start the service due to personal values about abortion or legal concerns. Additionally, only two of the pharmacists were found to be filling out the registration logbook chart. Among the pharmacists that were actively providing MA services, they were making use of both the Hesperian Safe Abortion App and distributing the IEC materials to their clients (Supplementary Material 1).

To build broader support for the service and address bottlenecks in the effort, the team of Ipas and Regional Health Bureau staff established a gap-filling support system for the trainees, the pharmacy owners, and other pharmacists on staff during the visit. Apart from the onsite support to resolve the challenges, Ipas provided each pharmacy with a start-up MA supply of ten combipacks (kits containing the recommend doses of mifepristone and misoprostol) to commence service immediately post-training. They additionally facilitated relationships between the trainees and MA suppliers to ensure a continuous supply of MA drugs going forward. This included consulting DKT and providing them with a list of the pilot pharmacies during this process to ensure that their MA supply needs were prioritized in case of any stock outs. For the remainder of the pilot period, joint supportive supervision visits were conducted by Ipas, the RHB, and City Health Offices quarterly to continuously fill the emerging gaps and gather insights for adapting the intervention.

### 3.2 Results of the pilot intervention

Out of the total 41 pharmacies included in the pilot, 32 provided MA drugs to people seeking abortion. The remaining 9 facilities withdrew from the pilot for various reasons; 3 of the pharmacies in the first round of training were found to be drug stores and were therefore ineligible to provide MA at the time; 5 pharmacy owners had withdrawn due to personal values; and one of the owners passed away immediately after the training.

Table 2 presents summary statistics from pharmacy self-managed abortion services logbooks. A total of 1,457 clients received MA self-care services from the 32 private pharmacies in (16 in Adama and 16 in Shashemene towns). Most MA clients 972 (71.4%) were between 20 and 29 years of age, and 11% of clients were less than 19 years of age. All clients had gestational ages less than 10 weeks by last menstrual period (LMP) at the time of service. Most (98.6%) used the combined regimen with mifepristone and misoprostol, and only 17 (1.2%) clients received misoprostol only. More than half of users (59.1%) obtained information about the availability of SMA through pharmacies from their friends and 15.5% from their husbands/partners.

**Table 2:** Summary statistics from pharmacy self-managed abortion services logbooks.

	Total Clients (*N* = 1,457)	
	N	%
Location of pharmacy		
Adama town	502	34
Shashemene town	955	66
Total clients per pharmacy		
Mean (SD)	47	54.8
Client successfully contacted after 21 days		
Yes	1,360	93.3
No	97	6.6
Client age		
≤16	15	1.1
17–19	135	9.9
20–24	595	43.7
25–29	377	27.7
30–34	124	9.1
≥35	32	2.3
Missing	82	6.0
MA Method Received		
Misoprostol only	17	1.2
Mifepristone and misoprostol	1,341	98.6
Missing	2	0.1
Source of information		
Friends	804	59.1
Family	114	8.3
Husband/partner	212	15.5
Media	38	2.7
Other	169	12.4
Missing	23	1.7
Client consented to share SMA outcomes with pharmacist		
Yes	1,233	90.6
No	127	9.3
Took the drugs as per the instruction		
Yes	1,228	99.5
No	5	0.4
Completed abortion		
Yes	1,212	98.3
No	10	0.8
Unsure	11	0.9
Experienced danger signs of a complication		
Severe abdominal pain	2	0.1
High-grade fever	1	0.07
Foul-smelling discharge	1	0.07

Most (99.6%) had taken drugs as per the provided instruction by the pharmacists; only three clients (0.4%) took only the first dose, one client took the two doses all at once, and a final client's actions are unknown. Nearly all (98.3%) successfully completed the abortion using the MA drugs secured from the pharmacy. Ten clients (0.8%) did not have a complete abortion, and 11 (0.8%) clients were unsure about the completion of abortion at the time of interview. Clients with an incomplete abortion were advised by pharmacists to seek extra care from a health facility, including nearby public and private facilities. Among them (*n* = 10), four clients (40%) went to public health facilities and completed their abortion using manual vacuum aspiration (MVA); two clients (20%) did the same at a private clinic; two clients (20%) reported that they didn't have any bleeding after taking the drugs and didn't do anything; and two clients (20%) still had bleeding and had not completed the abortion at the time of the call. Among all clients, only four (0.3%) reported that they were experiencing at least one of the danger signs of a complication; this included two clients with severe abdominal pain, and one client each with a high-grade fever or foul-smelling vaginal discharge, respectively.

Table 3 presents descriptive results from the randomly-selected 56 MA clients that participated in the structured survey administered by the M&E team. Results from the structured survey mirrored those from pharmacy logbooks. Almost all MA clients (98.2%) had taken the drug as per the counseling delivered by the pharmacist. Most clients (96.4%) had taken five pills in total, in line with the recommended regimen. All except one client successfully expelled their pregnancy in two doses. Only one client took an additional dose using misoprostol purchased from a different pharmacy to complete the termination. The follow-up survey also provided insights into the sociodemographic characteristics of clients. Among those sampled, client ages ranged from 18 to 35 years old with an average age of 24. They represented a diverse range of educational backgrounds, with the greatest proportion having completed secondary school (21.4%, *n* = 12). One-third were students (33.9%, *n* = 19), unemployed (26.8%, *n* = 15), or engaging in a small-scale business (21.4%, *n* = 12). Most clients were either unmarried (42.9%, *n* = 24) or living with a partner (25.0%, *n* = 14).

**Table 3:** Client and self-managed abortion procedure characteristics from follow-up questionnaire (*N* = 56).

	Total Clients (*N* = 56)	
	N	%
Client characteristics		
Age		
Mean (Min, Max)	24.4	(18, 35)
Highest educational attainment		
Never attended school	7	12.5
Completed some primary	11	19.6
Completed primary	7	12.5
Completed some secondary	10	17.9
Completed secondary	12	21.4
Attended vocational/college/university	9	16.1
Occupation		
Daily laborer	7	12.5
Professional/technical/managerial workers	3	5.4
Small-scale business	12	21.4
Student	19	33.9
Unemployed/dependent/housewife	15	26.8
Relationship status		
Currently married	8	14.3
Living with a partner	14	25
Never married	24	42.9
Separated/divorced	9	16.1
Refused to answer	1	1.8
Self-managed abortion procedure characteristics		
Total # of pills taken		
Median (Min, Max)	5	(1,6)
# pills in first dose (Mifepristone)		
Median (Min, Max)	1	(1,3)
Route of first dose		
Swallowed	53	94.6
Sublingual	3	5.4
Took second dose		
Yes	54	96.4
No*	2	3.6
# pills in second dose (Misoprostol)		
Median (Min, Max)	4	(2, 4)
Route of second dose		
Vaginal	45	83.3
Sublingual	9	16.7
Took the drugs as per the instruction		
Yes	55	98.2
No**	1	1.8
Needed additional treatment to complete abortion		
Yes	1	1.9
No	51	98.1

*Reasons for not taking second dose: already expelled products of conception (POC); already started bleeding.

**Reason for not following instructions: client could not remember what the pharmacist told them.

## 4 Discussion: practical implications and lessons learned

### 4.1 Pilot program successes

When considering abortion, many people turn to private pharmacies as their initial stop for information and counseling. Results from this pilot confirmed that demand for self-managed abortion through pharmacies is strong. In just fifteen months, 1,457 pregnant people sought SMA through intervention pharmacies in Adama and Shashemene towns. Nearly all (98.3%) had a complete abortion without needing additional treatment, and most of those who experienced an incomplete abortion successfully received a referral to additional services in a public or private health facility. This result is comparable to the 99% and 96% of clients reporting completed abortions without additional treatment documented in a community-agent SMA program in Bolivia (13) and an online US telemedicine program (14), respectively. Clients were able to distinguish between the expected side effects of MA and the dangers signs of a complication, and therefore only six clients sought PAC services through a health facility following their pharmacy visit. This is similar to the low levels of follow-up treatment required in non-inferiority evaluations comparing pharmacy and clinic-accessed MA in Ghana and Cambodia (15, 16). Women accessing MA through pharmacies in this pilot correctly followed the MA drug regimen that pharmacists counseled them on; in fact, our pilot program documented a comparatively high rate of adherence to the MA drug regimen (99.6% of clients) compared to 86.7% of pharmacy clients in a non-inferiority trial in Ghana (15). These results evince the pilot program's success in facilitating quality SMA counseling, information, and drugs through pharmacies. Despite these encouraging findings, the pilot project also experienced several challenges that should be known and used to inform future SMA interventions in Ethiopia and similar settings.

### 4.2 Pilot program challenges

Self-managed abortion is a recent trend in Ethiopia, and therefore there is an inadequate legal and structural framework to support SMA care-seekers and access points. Hence, various challenges were encountered at different levels: lack of a legal framework supporting SMA through pharmacies; weak private-public partnerships; pharmacist resistance or hesitance to participate; MA supply chain issues; and service-level barriers at pharmacies.

Although pregnant people continue to obtain MA through private pharmacies and other informal vendors, the legal system has not yet standardized the practice. In 2014, the Safe Abortion Care Technical and Procedural Guidelines was revised to reflect significant clinical updates, including expanded gestational limits for MA. However, the recommendation did not incorporate self-care as a part of the continuum of care for abortion. The lack of a legal framework to implement abortion self-care has made it difficult for the pharmacy owners and pharmacists to implement SMA without experiencing fear and hesitation due to perceived legal risks, even despite the pilot team's engagement with those regulatory bodies. This led to some initial resistance to be part of the pilot initiative and participate in the three-day training. We had difficulty finding pharmacies that openly admitted to dispensing the medication out of concern for possible legal implications. In a May 2023 review meeting with the SMA pilot stakeholders, one participant explained:

“The fear we had has limited our service provision during the implementation. Regardless of the information we were given during the training and supervisory visits, while providing the services in [town] we hesitate that we might be accused if something happened since we don’t have any written documents. Sometimes I decide to interrupt the service and pause for few days and again another time, I continue to provide the services. It would be better if we had a written document so that it helps us to build confidence.” [May 2023 SMA pilot review meeting report]

Some participants dropped out of the training in fear of being legally accountable, owing to religious beliefs, and some of them expressed resistance and skepticism about the aim of the intervention. Furthermore, we have observed that the values and support of owners, or even their spouses, were significant since in some instances, trainees were unable to resume service due to a lack of support from the owners.

During the pilot implementation, we learned that the pre-existing relationships between private sector pharmacies and the Zonal Health Offices were more focused on regulatory and control issues than on partnership. Ipas had worked closely with and supported the public health system to expand CAC through collaboration with the ministry and regional bureaus. However, Ipas's engagement with the private sector was limited, with no prior collaboration, particularly with private pharmacies in the region. These loose relationships posed a challenge at first, particularly in terms of trust among these three parties, which led to doubts about the intervention's intention and fear of potential accountability related risks during implementation. As a result, ongoing engagement and relationship building was critical to sustaining the service and eventually building trust.

Stockout of MA drugs was one of the main reasons for not providing the service especially after the first round of the training held in Adama. This was mainly due to limited availability of the drugs from suppliers following that training and some confusion between pharmacy owners, their staff, and Ipas about procurement protocols and responsibilities. In addition, in pharmacies where multiple staff work in shifts, having only one trained employee immediately limited the availability of the service to their shift or made it appear unavailable when they were not on duty. Moreover, the turnover of trained staff, personal value and commitment issues, and inconsistency in keeping records of the service were some of the challenges faced during service provision.

Some trained pharmacists also indicated that some of the clients had difficulty in understanding the instructions correctly mainly due to language barrier or low health literacy. Furthermore, pharmacists reported that some clients intentionally decreased their gestational age estimates out of fear that they would be turned away or referred elsewhere. As explained during the May 2023 review meeting:

“Some women decrease their gestational age during history taking and do not want to tell the exact pregnancy ages. After repeated counseling and discussion they tell us that they decrease because of not to be linked referred to other facilities. They did that just to get the services, hence counseling such clients is time consuming. Therefore, it requires the motivation and commitment of the providers.” [May 2023 SMA pilot review meeting report]

Finally, some pharmacists also reported difficulties when male partners requested MA drugs without their pregnant partner present, limiting the pharmacist's ability to properly assess and counsel the client.

### 4.3 Lessons to inform future applications of pharmacy-supported SMA

The lack of standardized service delivery at private pharmacies combined with the absence of legal frameworks and ever-rising demand for MA from these outlets poses a considerable challenge to the health and wellbeing of women and girls all across Ethiopia. At the same time, the situation also represents an opportunity to drastically improve access to quality abortion care via direct support to private pharmacies for SMA.

During the COVID pandemic, the Ministry of Health initiated a self-care guideline to strengthen the Regional Maternal, Newborn, Child, Adolescent, and Youth Health (RMNCAYH) services that have been adversely impacted by the pandemic. The implementers of this pilot have been actively engaged to incorporate abortion self-care and task shifting in the guideline. As a result, Ethiopia's National Self-Care Guideline has been recently updated and approved by the Ministry of Health to incorporate SMA (22). However, the new guidelines still require the support of a health care professional, despite WHO's guidance that this is not necessary. At the time of writing this paper, the updated guidelines had not yet been made available to the public, but official launch plans are underway.

It is crucial to strengthen and support these private pharmacies both programmatically and through appropriate legal frameworks, including the National Self-Care Guideline. Based on the SMA pilot results and the evidence generated so far, we recommend that private sector pharmacies, through adequate multi-sector support, can improve access to services for people seeking abortions and bring safe services closer to pregnant people. This requires a commitment to strengthening the referral network in these two cities and scaling up to other regions of the country, targeting both the public and private sectors for enhanced service provision, networking, and partnership. Consensus-building platforms must be set up between referral points (both private clinics and public health facilities), pharmacies, and government counterparts such as Zonal Health Offices to create a supportive and accountable health care system that responds to the needs and choices of pregnant people. Finally, it is imperative that all future efforts include strategies to influence the current legal landscape to build a legal framework that supports private pharmacies' critical role in safe abortion access, with or without the direct involvement of a healthcare provider.

## 5 Acknowledgement of methodological constraints

This case study has a few methodological constraints that must be noted. First, as a pilot program, sustainability of the intervention was not evaluated. We were also unable to evaluate whether the pilot program had an impact on the volume of postabortion complications presenting at surrounding public health facilities. Given the lack of a legal framework supporting pharmacies for self-care, it is unclear whether pharmacists would feel confident and willing to continue SMA services to the same extent after supportive supervision visits end. Second, pharmacists were limited in their ability to follow up with all women who purchased MA drugs. Due to gender norms and the stigmatized nature of abortion and women's sexuality, some clients provided fake phone numbers or changed their phone numbers after sharing it with pharmacists, presumably to protect their identities. Still others did not have access to a phone that only they could access; many shared a phone with their partners or family members. As a result, 11% of women did not consent to a follow-up call. Among those that did consent, 81 (11%) were unreachable at the phone number provided. This introduces potential response bias into the pilot results. Third, because SMA is done outside of a health facility setting, the pilot evaluation had to rely on self-reported outcomes and complications by clients in place of a clinical confirmation of pregnancy termination. This measurement approach has been adopted widely in SMA research and is an accepted limitation of this approach. Despite these methodological constraints, evidence from this pilot suggests that scale-up of the pharmacy-supported SMA model should be integrated into national programs that aim to increase the availability and accessibility of high-quality safe abortion care in Ethiopia.

## Data Availability

The original contributions presented in the study are included in the article/Supplementary Material, further inquiries can be directed to the corresponding author.

## Footnotes

^1^The recommended combined regimen for MA is a single 200mg oral dose of mifepristone (one pill), followed by a single 800mcg dose of misoprostol (four pills of 200mcg each, administered buccally, sublingually, or vaginally) one to two days later. The recommended misoprostol-only regimen is 800mcg (four pills) administered buccally, sublingually, or vaginally every 3 h until expulsion (19).

^2^Hesperian’s Safe Abortion App can be accessed at https://hesperian.org/safe-abortion-app/, in Google Play, and in the iPhone App Store.
