# Supplementary material for: Increasing access to self-managed abortion through pharmacies: programmatic results and lessons from a pilot program in Oromia, Ethiopia
Source: Front Reprod Health. 2025 Mar 4;7:1472696. doi: 10.3389/frph.2025.1472696 (PMC11917366; doi:10.3389/frph.2025.1472696)
Supplement: Supplementary file 1 [file Datasheet1.pdf]

Supplementary Material

Supplementary Figure 1. Brochures distributed by pharmacists to clients purchasing MA. Top: Side 1, Amharic (left) & Oromo (right). Bottom: Side 2, Amharic (left) & Oromo (right).

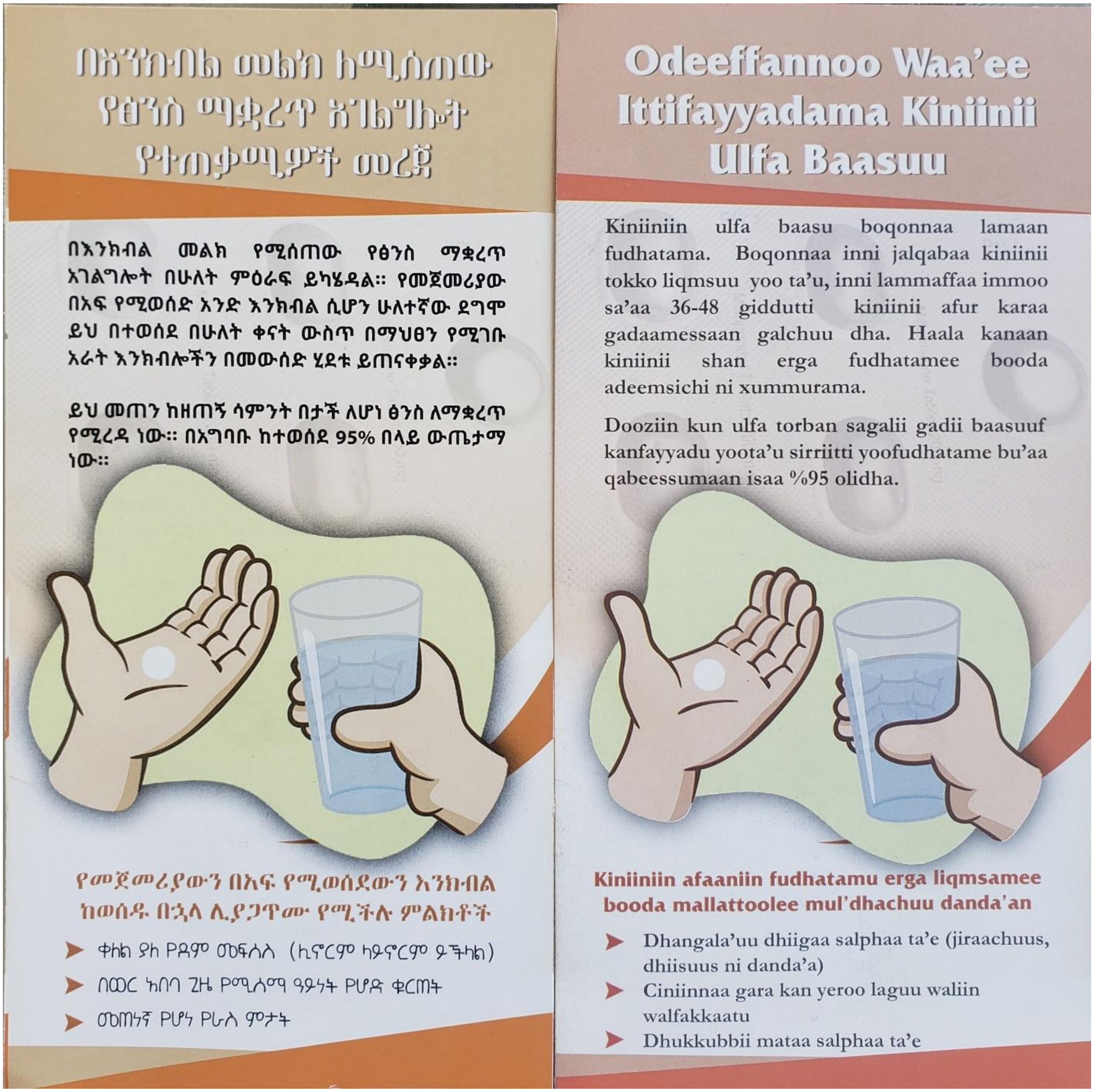

በማህፀን ውስጥ የሚገቡ 4 እንክብሎችን ከወሰዱ በኋላ የሚታዩ ምልክቶች

- የወር አበባን የሚያህል ወይም ከዛም የሚበዛ የደም መፍሰስ
- የረጋ ደም መፍሰስ
- የሆድ ቁርጠት

የፅንስ መቋረጥ ሂደቱ እስኪጠናቀቅ ድረስ በሚመችዎት ስፍራ ቢሆኑ ይመከራል። መደበኛ የሆነውን አመጋገብዎንም መከተል ይችላሉ።

የሚከተሉት ችግሮች ካጋጠምዎት ግን በአፋጣኝ ወደ ጤና ተቋም መሄድ ይኖርብዎታል

- ከፍተኛ የሆነ የደም መፍሰስ የሚኖር ከሆነ። በተለይም ከፍተኛ ድካምና እንቅልፍ እንቅልፍ የማለት ስሜት ከተሰማዎት
- ባለአራት እንክብል መድኃኒት ከወሰዱ በኋላ እረዘም ላለ ጊዜ ትኩሳት የሚሰማዎ ከሆነ
- ለረጅም ጊዜ የሚቆይ ተከታታይነት ያለው የሆድ ቁርጠት፣ የጠና ህመም እንዲሁም ከብልት የሚወጣ መጥፎ ሽታ ያለው ፈሳሽ ካለቦት
- ከፍተኛና ተከታታይነት ያለው ማስታወክ ካጋጠመዎ
- ከፍተኛና ተከታታይነት ያለው የራስ ምታት ካጋጠመዎ

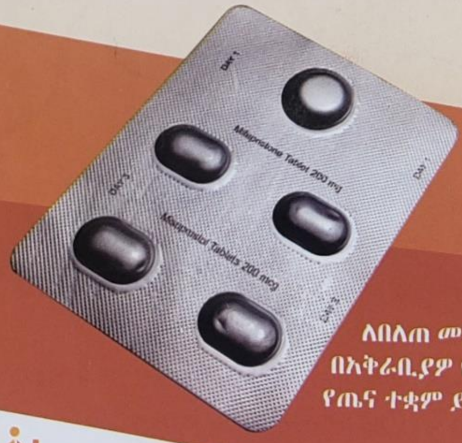

ለበለጠ መረጃ
በአቅራቢያዎ የሚገኝ
የጤና ተቋም ይጎብኙ።

Ipas
Health. Access. Rights.

Kiniiniiwwan 4 karaa gadaamessaa erga galfamanii booda mallattoolee mul'atan

- Dhangala'uu dhiigaa kan hanga yeroo laguu gahu ykn sana ol ta'u
- Dhangala'uu dhiga ititaa
- Ciniinnaa garaa

Haala asiin olitti ibsameen adeemsi ulfa baasuu hanga xummuramutti bakka isinitti tolu yoo turtan gaarii dha. Nyaata idilee keessanis ittifufuu dandeessu.

Rakkooleen asiin gaditti tarreefaman yoo mul'atan garuu hatattamaan gara dhaabbilee fayyaa deemuu qabdu.

- Dhiigni hedduun yoo kan dhanagala'u ta'e; keessattuu dadhabbii fi miirri hirribaa yoo isinitti dhaga'ame
- Erga kiniiiniiwwan arfan fudhattanii booda, gubiinsi qaamaa yoo isin muudate.
- Ciniinnaa garaa yeroo dheeraa, dhukkubbii cimaa fi yaa'aan foolii gaarii hinqabne kara qaama wal hormaataa yoo jiraata.
- Hoqqisiisaa cimaa fi deddeebi'aan yoo isin muudate
- Mataa bowwoo cimaa fi yeroo dheeraaf turu yoo isin muudate

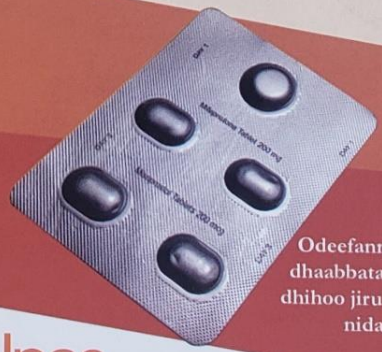

Odeefannoo dabalataa dhaabbata fayyaa isinitti dhihoo jiru irraa argachuu nidandeessu

Ipas
Health. Access. Rights.
